# Computed tomography-based body composition parameters can predict short-term prognosis in ulcerative colitis patients

**DOI:** 10.1186/s13244-024-01615-w

**Published:** 2024-02-27

**Authors:** Jun Lu, Hui Xu, Haiyun Shi, Jing Zheng, Tianxin Cheng, Minsi Zhou, Xinjun Han, Yuxin Wang, Xuxu Meng, Xiaoyang Li, Jiahui Jiang, Peng Li, Zhenghan Yang, Lixue Xu

**Affiliations:** 1 Department of Radiology, Beijing Friendship Hospital, Capital Medical University, No. 95 Yongan Road, Beijing, 100050 China; 2 Department of Gastroenterology, Beijing Friendship Hospital, Capital Medical University, No. 95 Yongan Road, Beijing, 100050 China

**Keywords:** Ulcerative colitis, Computed tomography, Body composition parameters, Short-term prognosis

## Abstract

**Objectives:**

Emerging evidence suggests a potential relationship between body composition and short-term prognosis of ulcerative colitis (UC). Early and accurate assessment of rapid remission based on conventional therapy via abdominal computed tomography (CT) images has rarely been investigated. This study aimed to build a prediction model using CT-based body composition parameters for UC risk stratification.

**Methods:**

In total, 138 patients with abdominal CT images were enrolled. Eleven quantitative parameters related to body composition involving skeletal muscle mass, visceral adipose tissue (VAT), and subcutaneous adipose tissue (SAT) were measured and calculated using a semi-automated segmentation method. A prediction model was established with significant parameters using a multivariable logistic regression. The receiver operating characteristic (ROC) curve was plotted to evaluate prediction performance. Subgroup analyses were implemented to evaluate the diagnostic efficiency of the prediction model between different disease locations, centers, and CT scanners. The Delong test was used for statistical comparison of ROC curves.

**Results:**

VAT density, SAT density, gender, and visceral obesity were significantly statistically different between remission and invalidation groups (all *p* < 0.05). The accuracy, sensitivity, specificity, and area under the ROC curve (AUC) of the prediction model were 82.61%, 95.45%, 69.89%, and 0.855 (0.792–0.917), respectively. The positive predictive value and negative predictive value were 70.79% and 93.88%, respectively. No significant differences in the AUC of the prediction model were found in different subgroups (all *p* > 0.05).

**Conclusions:**

The predicting model constructed with CT-based body composition parameters is a potential non-invasive approach for short-term prognosis identification and risk stratification. Additionally, VAT density was an independent predictor for escalating therapeutic regimens in UC cohorts.

**Critical relevance statement:**

The CT images were used for evaluating body composition and risk stratification of ulcerative colitis patients, and a potential non-invasive prediction model was constructed to identify non-responders with conventional therapy for making therapeutic regimens timely and accurately.

**Key points:**

• CT-based prediction models help divide patients into invalidation and remission groups in UC.

• Results of the subgroup analysis confirmed the stability of the prediction model with a high AUC (all > 0.820).

• The visceral adipose tissue density was an independent predictor of bad short-term prognosis in UC.

**Graphical Abstract:**

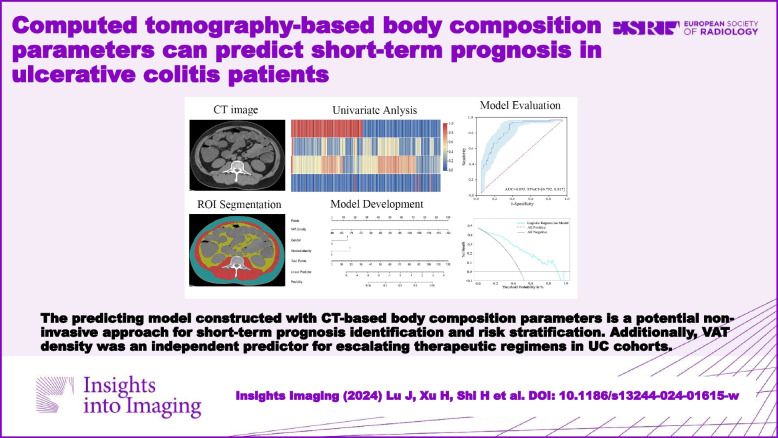

**Supplementary Information:**

The online version contains supplementary material available at 10.1186/s13244-024-01615-w.

## Introduction

Ulcerative colitis (UC) is a chronic inflammatory bowel disease (IBD) diagnosed by considering clinical manifestations, laboratory tests, imaging analysis, and histological criteria [1, 2]. The goal of treatment of UC is to improve health-related quality of life and avoid disability [3]. Therefore, it is important to achieve rapid relief of clinical symptoms in the short term (within 6 months) and to achieve endoscopic healing when possible [4]. Traditional conventional treatments such as 5-aminosalicylates [5-ASA], corticosteroids, and thiopurine immunomodulators have been widely used and have shown high efficacy in achieving clinical remission [1]. Although traditional conventional therapy is in accordance with relevant guidelines for treating UC, the treatment response varies in different patients. Approximately 30 to 40% of mild-moderate patients cannot achieve rapid clinical relief through conventional therapy and predicting outcome is difficult [5]. A critical issue in clinical treatment is to determine the exact time to escalate treatment if patients fail to achieve rapid remission from conventional therapy. While several clinical laboratory indicators such as C-reactive protein (CRP) and erythrocyte sedimentation rate (ESR) can reflect the severity of the disease to some extent, they fail to identify those who cannot achieve rapid relief. Fecal calprotectin (FC) is correlated with histological activity and is expected to be a surrogate non-invasive marker for histological assessment [6]. However, due to limited healthcare resources and economic considerations, FC assay was not routinely performed in many hospitals. Emerging evidence suggests a link between body composition and the illness behavior of UC, which can be assessed by standard abdominal computed tomography (CT) [7]. Previous studies have revealed the correlations between certain body compositions such as visceral adipose tissue and skeletal muscle and outcomes of IBD [8, 9]. Understanding the relationship between body composition and the short-term prognosis of UC could improve our understanding of non-response to conventional therapy and aid in better positioning and escalating treatment strategies of available agents. However, little is known regarding the association between body composition and UC, and it is urgent to determine which body composition parameter was related to the short-term prognosis of UC during clinical practice.

Besides, intestinal inflammation might induce dynamic changes in the balance between water and lipid content in body composition, especially the visceral and subcutaneous adipose tissue. However, the imaging information was underutilized. There were few studies to analyze the dynamic changes captured by the CT attenuation index. More importantly, achieving rapid relief of clinical symptoms or endoscopic healing could contribute to improving quality of life and avoiding long-term poor prognosis [1, 3]. Therefore, there is an urgent need for more comprehensive and definitive clinical predictors for risk stratification patients with UC and to make a tailored and upgrading treatment plan timely and early. Thus, this study aimed to find effective CT-based body composition parameters for non-invasively identifying the patients who cannot achieve rapid remission from conventional therapy at the initial stage of diagnosis.

## Methods

### Patients and study design

This retrospective study was approved by the Institutional Review Board, and the requirement for informed consent was waived. From April 2017 to June 2022, the patients who were first diagnosed with UC were consecutively enrolled in two centers. The inclusion criteria were as follows: [a] adults with a definite diagnosis of UC based on clinical manifestations, endoscopic, imaging analysis, and histological criteria in line with the World Gastroenterology Organization Global Guidelines; [b] available abdominal CT within 1 week before starting treatment; [c] available complete clinical data (age, gender, height, weight, smoking status, location of disease, CRP, ESR, albumin, neutrophil count, lymphocyte count, and modified Mayo Score); and [d] at least 6 months follow-up after accepting conventional therapy unless they underwent surgery or switched to biologic agents due to basic drug failure within 6 months. The exclusion criteria were as follows: [a] poor image quality with severe motion artifact, [b] patients with a history of abdominal surgery within 3 months before the CT scan, and [c] along with malignant tumor or severe organ dysfunction.

### Follow-up and outcomes

All the treatments for the patients followed the relevant guidelines and regulations, using a joint doctor-patient decision-making approach to make a treatment plan. The conventional therapy was referred to well-established traditional treatment plan such as 5-aminosalicylates [5-ASA], corticosteroids, and thiopurine immunomodulators.

The clinical data such as CRP, ESR, and modified Mayo (mMayo) score were collected at the first admission and every 3–4 months during the 6-month follow-up period. The patients were considered to achieve rapid remission at a definite time point of 6 months after conventional therapy initiation, which meets one of the following criteria: clinical or endoscopic response according to the mMayo score. If not, the treatment was deemed to be invalid (bad short-term prognosis). The mMayo score comprises three subscores: endoscopic findings, stool frequency, and rectal bleeding, as recommended by the US Food and Drug Administration (FDA) [10]. Clinical response was defined as rapid relief of clinical symptoms including stool frequency and rectal bleeding. The specific criteria were a stool frequency subscore of ≤ 1 point, a decrease in rectal bleeding subscore of ≥ 1 point, or an absolute rectal bleeding subscore of 0 or 1. The endoscopic response was defined as endoscopic findings subscore of 0 or 1. If patients underwent surgery or switched to biologic agents, they were regarded as non-responders.

### CT image analysis

#### Slice selection

The computed tomography (CT) images were reviewed by a radiologist (with 5 years of experience in abdominal CT). The specific slices were defined according to the location of the disease. If the disease (proctosigmoiditis) is limited to the rectum and a portion of the sigmoid colon, the level of the third vertebra (L3) was chosen to define the region of interest (ROI). That is to say, the slice of L3 was segmented to obtain body composition parameters if the lesions were below the level of the fifth vertebra (L5) as previous studies did [7, 11]. If the disease extends from distal to splenic flexure or proximal to the splenic flexure (left-side colitis or pancolitis), the level with the most significant thickened intestinal wall was chosen to define the ROI. The lesions were almost located at the level of the second to the fourth vertebra (L2–4) in this study (Additional file 1: Table S1).

#### Assessment of body composition

**Fig. 1:**
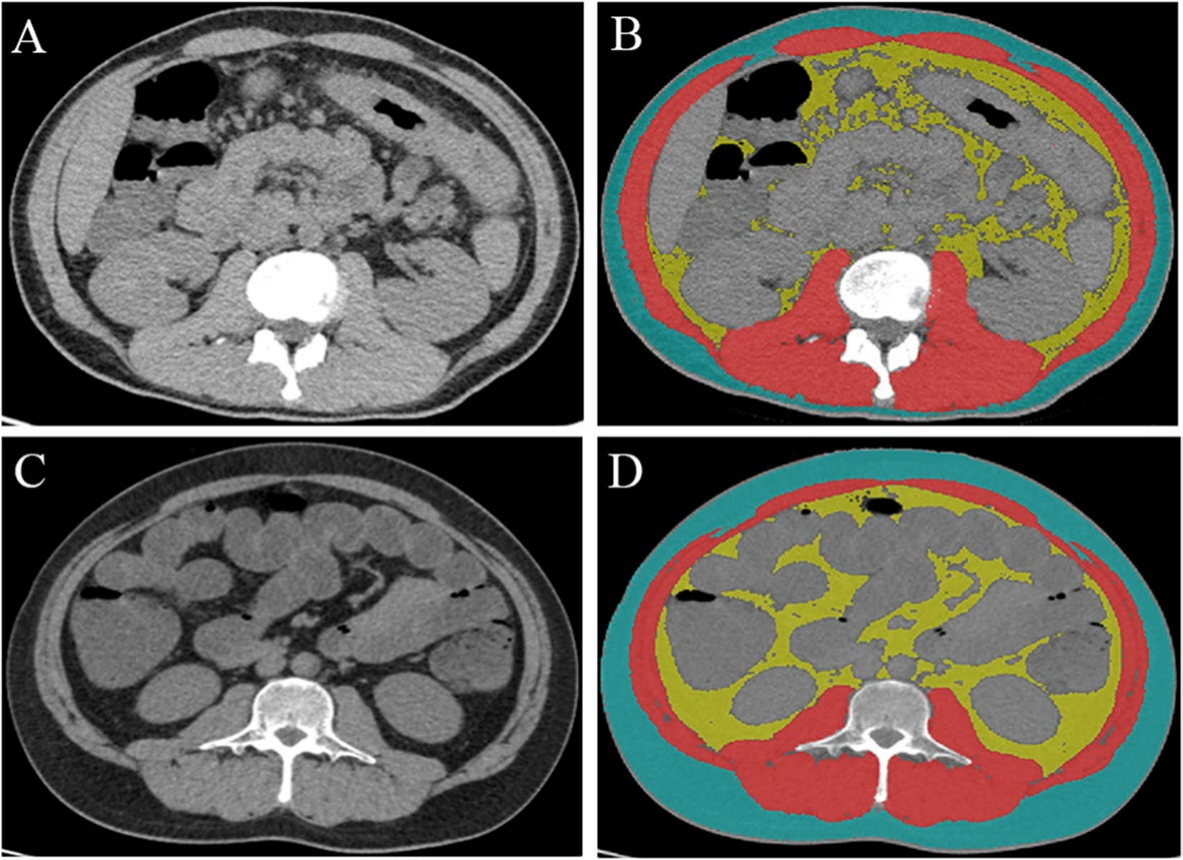
**A** Level of the second vertebra (L2) was selected with the significant thickened intestinal wall in the transverse colon. **B** The results of semi-automated segmentation. **C** Level of the third vertebra (L3) was selected if the disease was limited to the rectum. **D** The results of semi-automated segmentation. The blue area represents the subcutaneous adipose tissue. The red area represents the skeletal muscle. The yellow area represents the visceral adipose tissue

All patients underwent abdominal CT using one of the four CT scanners (Additional file 1: Table S2) in two centers. The skeletal muscle and adipose tissue were segmented on non-contrast abdominal CT by two radiologists (Z.J. and L.J., with 2 and 5 years of experience in abdominal CT, respectively) as previous studies did [12]. The segmentation was carried out using slice-O-matic (version 5.0, TomoVision 3280 Ch, Canada) with a semi-automated segmentation method. The skeletal muscle, visceral adipose tissue (VAT), and subcutaneous adipose tissue (SAT) were defined with attenuation thresholds -29 to 150 HU, -50 to -150 HU, and -30 to -190 HU, respectively (Fig. 1). The area (cm^2^) and mean CT attenuation index (HU) were measured according to the results of semi-automated segmentation. One radiologist (L.J.) repeated the semi-automated segmentation and measurement after 1 month. Skeletal muscle index (SMI) was defined as the skeletal muscle area (cm^2^) divided by height squared (m^2^). Similarly, visceral adipose index (VAI) and subcutaneous adipose index (SAI) were defined as the visceral adipose tissue area and subcutaneous adipose tissue area divided by height squared. Several classification indicators based on body composition were defined. Sarcopenia was defined as SMI ≤ 50 cm^2/^m^2^ in men and SMI ≤ 39 cm^2/^m^2^ in women [13]. Sarcopenic obesity was defined as concurrent obesity (BMI ≥ 30 kg/m^2^) and sarcopenia [12]. Visceral obesity was defined as the area of VAT area ≥ 130 cm^2^ as the previous study did [14]. Additionally, the ratio of visceral to subcutaneous adipose tissue area (VSR) and the ratio of visceral to total adipose tissue (visceral and subcutaneous fat) area (VTR) were calculated.

### Prediction model development and evaluation

Variables significant in the univariate analysis were included to build a predicting model with a multivariable logistic regression. The diagnostic performance, calibration, and clinical utility of the prediction model were evaluated by using the receiver operating characteristic (ROC) curve, calibration curve, and decision curve analysis (DCA), respectively. In the framework of the DCA result, the clinical net benefit of the model was calculated for each possible threshold probability by summing the benefits (proportion of true-positive) and subtracting the harms (proportion of false-positive), which puts benefits and harms on the same scale [15].

Besides, stratification analysis related to predicting the model’s diagnostic performance was performed. Given that ulcerative colitis can be found in different locations, evaluation of the prediction model’s ability to identify bad short-term prognosis in different disease locations is necessary. Thus, the diagnostic performance of the prediction model was compared among three different disease locations. We also performed subgroup analysis to evaluate the diagnostic efficiency of the prediction model and its stability and robustness between different centers and different CT scanners.

### Statistical analysis

Intra- and interclass correlation coefficient (ICC) analyses were performed to evaluate the reproducibility and stability of body composition parameters from CT images. The correlation among parameters was analyzed by the Spearman correlation. The Mann–Whitney *U* test or independent *t*-test was used for continuous variables. Differences in categorical variables were analyzed using the chi-squared test or Fisher’s exact test. ROC curve was plotted to evaluate the prediction performance of the model. The Delong method was used for statistical comparison of the ROC curves. The calibration curve (bootstrap, *n* = 1000) and the Hosmer–Lemeshow test were used to evaluate the calibration of the prediction model. Decision curve analysis (DCA) was applied to reflect the clinical utility of the prediction model. The *p* < 0.05 was considered statistically significant.

## Results

### Patient characteristics

A total of 138 patients (107 from center 1 and 31 from center 2) were enrolled in two centers. Sixty-six (47.83%) achieved clinical or endoscopic remission after conventional therapy and 72 (52.17%) did not. The baseline characteristics of the 138 UC patients between the two groups are shown in Table 1. Only gender was significantly statistically different. Of the 72 who did not achieve remission, 25 switched to biologic agents (17 vedolizumab, 6 infliximab, 2 adalimumab) within 6 months. With the upgraded treatment plan, 13 patients achieved endoscopic response and 9 achieved clinical response. Only 2 patients did not achieve remission, and 1 patient did not follow up regularly.

**Table 1:** Patient baseline characteristics

Characteristics	Total (*n* = 138)		*t*/${x}^{2}$	*p*
	Remission (*n* = 66)	Invalidation (*n* = 72)		
Age (years)	48.32 ± 15.41	45.90 ± 15.07	0.930^a^	0.354
Gender			6.433^b^	0.011
Female	27	45		
Male	39	27		
Height (m)	1.66 ± 0.08	1.68 ± 0.07	1.650^a^	0.101
Weight (kg)	61.14 ± 13.73	62.96 ± 12.29	0.822^a^	0.412
BMI (kg/m^2^)	22.01 ± 4.14	22.12 ± 3.85	0.158^a^	0.874
Smoking status			1.156^b^	0.561
Never	49	52		
Previous	11	16		
Current	6	4		
Location of disease			3.410^b^	0.182
E1	8	5		
E2	24	19		
E3	34	48		
ESR	30.06 ± 21.60	35.33 ± 26.58	-1.272^a^	0.206
CRP	25.25 ± 35.42	29.71 ± 38.66	-0.705^a^	0.482
Albumin	33.62 ± 5.37	33.41 ± 5.27	0.229^a^	0.819
Neutrophil count	5.24 ± 2.94	4.86 ± 2.55	0.811^a^	0.419
Lymphocyte count	1.67 ± 0.56	1.65 ± 0.78	0.168^a^	0.867
Medication			4.817^b^	0.090
5-ASA	56	52		
5-ASA + immunomodulators	6	5		
5-ASA + corticosteroids	15	4		
Disease duration			3.763^b^	0.152
< 2 years	33	27		
2–5 years	10	20		
5 years	23	25		

*BMI* body mass index, *E1* proctosigmoiditis, *E2* left-side colitis, *E3* pancolitis, *CRP* C-reactive protein, *ESR* erythrocyte sedimentation rate, *5-ASA* 5-aminosalicylates

^a^Independent *t* test (*t*)

^b^Chi-square test (*x*^2^)

### Quantitative indicators based on body composition

Eleven quantitative parameters related to body composition were obtained with a semi-automated segmentation method. The ICC of these parameters was all ≥ 0.90 (Additional file 1: Table S3). In univariate analysis, only VAT density and SAT density showed statistical differences between the two groups (Table 2). Besides, the VAT density was highly correlated with SAT density (*r* = 0.809, *p* < 0.001). There were moderate correlations between VAT density and endoscopic Mayo score as well as mMayo score (*r* = 0.699, 0.536, respectively, all *p* < 0.001). Similar moderate correlations were observed between SAT density and corresponding Mayo score (*r* = 0.640, 0.549, respectively, all *p* < 0.001).

**Table 2:** Comparison of body composition parameters from abdominal CT images

Imaging parameters	Total (*n* = 138)		*t*	*p*
	Remission (*n* = 66)	Invalidation (*n* = 72)		
SMA surface	105.97 ± 30.44	105.19 ± 25.32	0.164^a^	0.870
VAT surface	84.80 ± 71.78	87.52 ± 64.46	-0.234^a^	0.815
SAT surface	114.24 ± 68.06	108.51 ± 80.05	0.451^a^	0.653
SMI	37.91 ± 8.74	36.75 ± 7.21	0.850^a^	0.397
VAI	30.07 ± 24.01	30.89 ± 22.96	-0.206^a^	0.837
SAI	41.49 ± 24.75	38.76 ± 29.59	0.586^a^	0.559
Muscle density	39.49 ± 8.99	41.02 ± 7.61	-1.076^a^	0.284
VAT density	-97.25 ± 6.53	-86.14 ± 10.70	-7.277^a^	< 0.001
SAT density	-98.98 ± 8.63	-88.13 ± 12.39	-5.918^a^	< 0.001
VSR (VAT/SAT ratio)	0.77 ± 0.54	0.89 ± 0.61	-1.237^a^	0.218
VTR (VAT/VAT + SAT)	0.39 ± 0.14	0.43 ± 0.15	-1.286^a^	0.200

*CT* computed tomography, *SMA* area of the skeletal muscle, *VAT* visceral adipose tissue, *SAT* subcutaneous adipose tissue, *SMI* skeletal muscle index, *VAI* visceral adipose tissue index, *SAI* subcutaneous adipose tissue index

^a^Independent *t* test (*t*)

### Classification indicators based on body composition

**Fig. 2:**
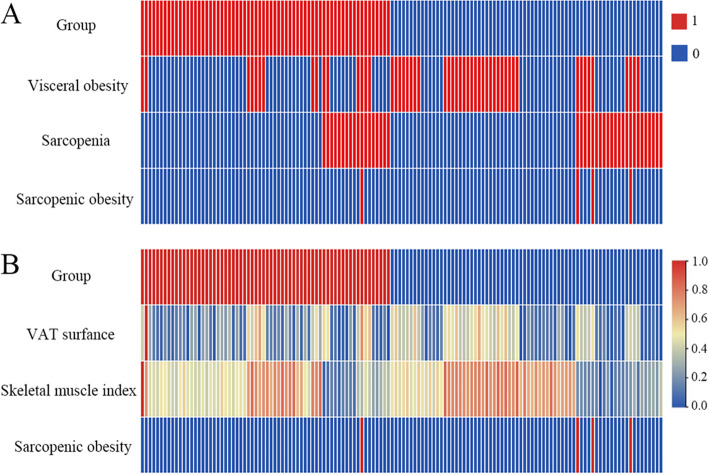
Heat map shows the distribution of body composition classification indicators between the two groups. Each row in the heat map corresponds to a unique feature and each column corresponds to one patient. **A** Group (1, remission; 0 invalidation), visceral obesity, sarcopenia, and sarcopenic (1, yes; 0, no). **B** Visceral adipose tissue (VAT) surface and skeletal muscle index (SMI) were continuous variables which were standardized from 0 to 1

Classification indicators from 138 UC patients are shown in Fig. 2. Fifty-two (37.68%) met the definition of visceral obesity. The distribution proportion of visceral obesity between the two groups (achieve remission and invalidation) showed a statistically significant difference. Forty-one (29.71%) and four (2.90%) met the definition of sarcopenia and sarcopenic obesity. The distribution proportion of the two indicators was not statistically different between the two groups.

### Construction and evaluation of the prediction model

Variables with significant group differences in the univariate analysis such as VAT density, SAT density, gender, and visceral obesity were included to build a predicting model with a multivariable logistic regression (backward LR). The final prediction model was as follows:

$$y=-0.149\times\mathrm{VAT}\;\mathrm{density}-1.192\times\mathrm{gender}+1.389\times\mathrm{visceral}\;\mathrm{obesity}-14.045$$

**Fig. 3:**
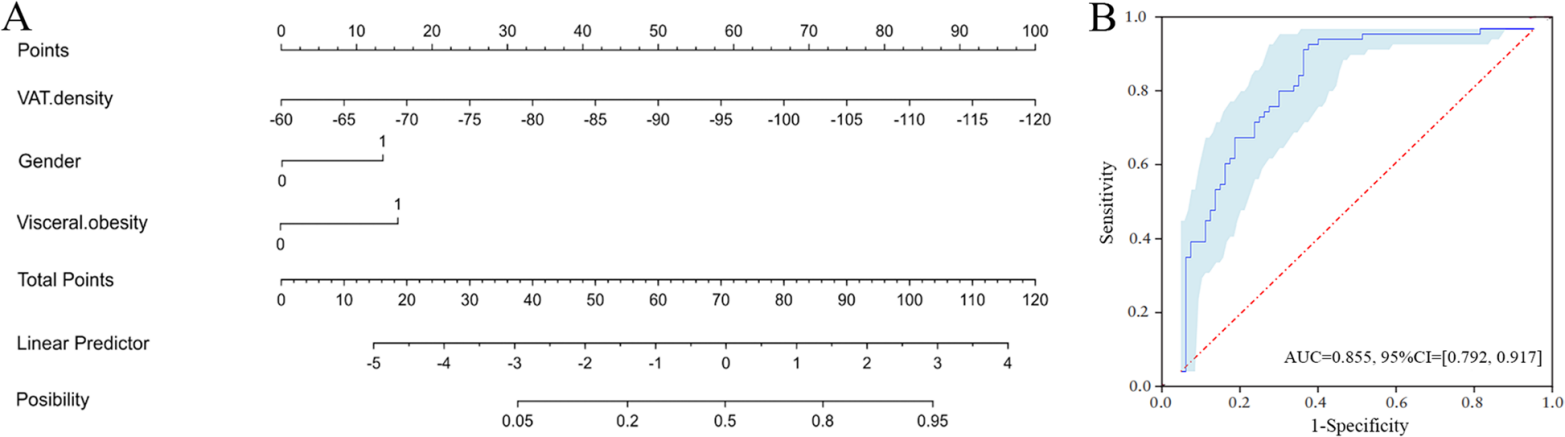
**A** The nomogram including VAT density, gender, and visceral obesity was plotted to represent the predicting model. **B** ROC curve of the predicting model for identifying the patients who cannot achieve rapid remission from conventional therapy

**Fig. 4:**
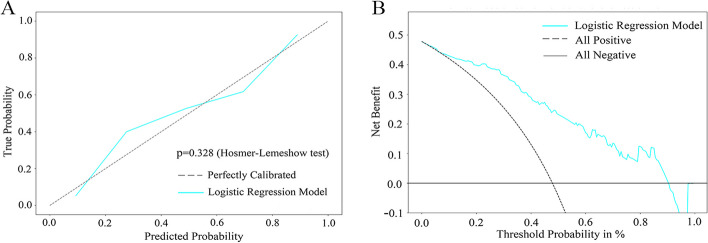
**A** Calibration curve of the predicting model showing that there was no difference between true probability and predicted probability (*p* = 0.328). **B** Decision curve analysis for the predicting model

Besides, a nomogram was plotted to represent the predicting model (Fig. 3A). The accuracy, sensitivity, specificity, and AUC of the prediction model were 82.61%, 95.45%, 69.89%, and 0.855 (0.792–0.917), respectively (Fig. 3B). The calibration curve and Hosmer–Lemeshow test concluded that the prediction model showed goodness of fit (*p* = 0.328), which was showed in Fig. 4A. The decision curve analysis showed that the prediction model could add clinical net benefit for identifying patients who could not achieve rapid remission from conventional therapy (Fig. 4B). Additionally, VAT density was an independent predictor for identifying the patients who cannot achieve remission.

### Diagnostic performances of the prediction model in different subgroups

In subgroup analysis, the VAT density and SAT density still showed statistical differences between the remission and invalidation groups in different disease locations (E1, E2, E3), different centers (center 1, center 2), and different CT scanners (CT1, CT2, CT3, CT4) (Table 3).

**Table 3:** Comparison of VAT density and SAT density in different subgroups

Subgroups	Imaging parameters	Total (*n* = 138)		*t*	*p*
		Remission	Invalidation		
E1 (8^a^:5^b^)	VAT density	-97.49 ± 6.28	-76.17 ± 16.11	-3.422^c^	0.006
	SAT density	-99.01 ± 6.69	-74.09 ± 18.81	-3.487^c^	0.005
E2 (24^a^:19^b^)	VAT density	-96.61 ± 6.25	-86.92 ± 11.56	-3.480^c^	0.001
	SAT density	-99.24 ± 7.62	-90.16 ± 11.31	-3.119^c^	0.003
E3 (34^a^:48^b^)	VAT density	-97.61 ± 6.91	-86.87 ± 9.35	-5.725^c^	< 0.001
	SAT density	-98.81 ± 9.78	-88.75 ± 11.39	-4.192^c^	< 0.001
Center 1 (53^a^:54^b^)	VAT density	-95.59 ± 8.42	-85.11 ± 11.90	-2.715^c^	0.011
	SAT density	-96.49 ± 8.48	-87.07 ± 11.91	-2.435^c^	0.021
Center 2 (13^a^:18^b^)	VAT density	-97.66 ± 6.01	-86.48 ± 10.37	-6.799^c^	< 0.001
	SAT density	-99.59 ± 8.64	-88.48 ± 12.64	-5.299^c^	< 0.001
CT1 (8^a^:4^b^)	VAT density	-98.06 ± 5.22	-84.53 ± 8.28	-3.509^c^	0.006
	SAT density	-102.31 ± 3.73	-88.02 ± 6.22	-5.05^c^	< 0.001
CT2 (17^a^:20^b^)	VAT density	-96.61 ± 5.33	-87.73 ± 8.49	-3.729^c^	0.001
	SAT density	-97.58 ± 7.25	-91.19 ± 7.82	-2.563^c^	0.015
CT3 (28^a^:30^b^)	VAT density	-98.18 ± 6.69	-85.92 ± 11.85	-4.807^c^	< 0.001
	SAT density	-100.05 ± 10.20	-86.74 ± 15.49	-3.833^c^	< 0.001
CT4 (13^a^:18^b^)	VAT density	-95.59 ± 8.43	-85.11 ± 11.91	-2.175^c^	0.011
	SAT density	-96.49 ± 8.48	-87.07 ± 11.91	-2.435^c^	0.021

*VAT* visceral adipose tissue, *SAT* subcutaneous adipose tissue, *E1* proctosigmoiditis, *E2 *left-side colitis, *E3* pancolitis

^a^The number of patients in the remission group

^b^The number of patients in the invalidation group

^c^Independent *t* test (*t*)

The diagnostic efficiency of the prediction model was evaluated in different subgroups.

In subgroup analysis, no significant differences in the AUC of the prediction model for identifying the patients who cannot achieve remission were found between different disease locations, different centers, and different CT scanners (all *p* > 0.05 after Bonferroni correction, Additional file 1: Table S4).

## Discussion

This study identified an association between CT-based body composition parameters and the short-term prognosis of UC. A prediction model including VAT density, visceral obesity, and gender can predict the bad short-term prognosis effectively. Importantly, VAT density was an independent predictor of patients who needed therapeutic regimen upgrades in our UC cohorts. Patients who failed to achieve rapid remission were inclined to exhibit a condition of visceral obesity.

At present, a growing number of studies have taken notice of the relationship between visceral adipose tissue and clinical outcomes of UC. However, the conclusions regarding the influence of VAT on IBD patients remained inconsistent. Kelly et al. concluded that visceral adiposity was not a predictor of the severity of inflammation or clinical course of acute severe UC [8]. In our study, the VAT surface also showed no statistical difference between the groups with rapid remission or not. A subsequent study with a larger cohort of Crohn’s disease (CD) showed that VAT density was closely related to the activity of inflammation [16]. Our study found that VAT density showed a moderate correlation with the activity of inflammation (mMayo score), too. Besides, patients who failed to achieve rapid remission were inclined to exhibit a higher VAT density. Finally, we found that VAT density is an independent predictor of non-responders UC patients who could not achieve rapid remission from conventional therapy. We suspect that it might attribute to an inflammatory reaction. Previous studies concluded that VAT plays an active role in systemic inflammation with the production of pro-inflammatory cytokines such as TNF-α and interleukin (IL)-6 [17]. Bilski et al. also thought that the visceral adipose compartment is not only a storage organ but also an endocrine organ, which is a possible source of pro-inflammatory substances [18, 19]. On the one hand, amounts of pro-inflammatory substances can directly or indirectly lead to a phenomenon called “leaky gut” along with disruption of the intestinal mucosa, an increase of intestinal permeability, and bacterial translocation [11, 20–22]. On the other hand, this phenomenon can aggravate the inflammatory reaction of adipose tissue. Finally, this excessive immune response reflected by dynamic changes in the balance between water and lipid content in body composition caused higher VAT density. In this study, the CT density changes of SAT and VAT were relatively consistent. But VAT density was the only independent predictor for identifying the patients who failed to achieve rapid remission. It is supposed that impaired mesenteric lymphatic drainage could also favor mesenteric adipocyte hyperplasia and increase fluid volume and edema, which could only be observed in VAT [23, 24].

Previous studies have shown that body composition parameters were promising for differential diagnosis of IBD [25], evaluating disease activity and severity of inflammation [26] and predicting adverse outcomes and recurrence [27, 28]. The above findings support efforts to develop a means of assessing the treatment outcomes. Thus, this study constructed a predicting model for non-invasively identifying those who could not achieve rapid remission from conventional therapy at the initial stage of diagnosis, which contributes to making a tailored and upgrading treatment plan timely and early for improving prognosis. Given that various CT scanners and scanning protocols exist in different centers, assessing CT parameters’ robustness is key to enabling its widespread use. Here, although the abdominal CT images were obtained from four different CT scanners with respective imaging parameters in two centers, our CT parameters still showed remarkable robustness. In different subgroup analyses, the accuracy of the prediction model for identifying the patients who cannot achieve rapid remission showed no significant statistical difference. The diagnostic performance of the prediction model in each center nearly matched the overall performance. As Li et al. constructed a reliable and stable CT-based prediction model for characterization of intestinal fibrosis [29], our CT-based prediction model had good generalization for identifying bad short-term prognosis. Of note, the difference of VAT density between the two groups was not affected by the different disease locations. The patients who failed to achieve rapid remission were inclined to exhibit a higher VAT density in every subgroup with E1, E2, and E3. In a word, the prediction model was of paramount importance to identify the bad short-term prognosis of UC. This CT-based non-invasive prediction method has attractive application prospects and can benefit patients of UC with an optimum therapeutic regimen at the initial stage of diagnosis.

Sarcopenia is prevalent and promising for predicting the prognosis of many chronic diseases such as cirrhosis [30], chronic kidney disease [31], congestive heart failure [32], and chronic obstructive pulmonary disease [33]. Previous study also confirmed that sarcopenia was associated with systemic inflammation as evidenced by elevated levels of TNF-α and IL-6 [34]. However, there were limited studies analyzing the prevalence and impact of sarcopenia on patients with UC. Zhang et al.’s study [35] demonstrated that the incidence of sarcopenia in UC was 27.3%, which was close to the result in our study of 29.7% (41/138). However, the results of our study showed that sarcopenia was not a significant predictor for bad short-term prognosis in UC patients.

There were several limitations in this study. Firstly, this was a retrospective study, and the selection bias was inevitable. Therefore, the significance of each parameter should be confirmed in a prospective multicenter study. Secondly, this was an exploratory analysis aiming to build a prediction model. It is also important to consider in a future longitudinal study whether the body composition parameters are different between the active and stable stages.

In conclusion, the predicting model constructed with CT-based body composition parameters is a potential non-invasive approach to risk stratification and early identification of patients who cannot achieve rapid remission from conventional therapy, and the CT-based body composition parameters showed robustness across different CT scanners. Additionally, VAT density was an independent predictor of the need for therapeutic regimen upgrades in UC cohorts.

### Supplementary Information

**Additional file 1: Table S1.** The specific number of patients about the different levels of vertebra chosen to define the region of interest (ROI) across different disease locations. L2, L3 and L4: the second, third and fourth level of vertebra; E1: proctosigmoiditis; E2: left-side colitis; E3: pancolitis. **Table S2.** The scanner parameters of the four computed tomography scanners. **Table S3.** The intra- and interclass correlation coefficient of computed tomography-based body composition parameters. SMA: area of skeletal muscle; VAT: visceral adipose tissue; SAT: subcutaneous adipose tissue; ICC: intra- and interclass correlation coefficient. The interval between time 1 and time 2 is one month. **Table S4.** Diagnostic performance of the prediction model to identify patients who could not achieve rapid remission from conventional therapy in different subgroups. E1: proctosigmoiditis; E2: left-side colitis; E3: pancolitis. Acc: accuracy; Sen: sensitivity; Spe: specificity; 95% CI: 95% confidence interval. The *p* value is the significance level of comparison of the AUC with that of random case (AUC = 0.05). The *p* < 0.05 was identified to be of statistically significance. Numbers in the parentheses represent the number of corresponding patients. **Fig. S1.** Flow chart of patients’ enrollment. UC: ulcerative colitis.

## Data Availability

The dataset used or analyzed during the current study are available from the corresponding authors upon reasonable request.

## Abbreviations

CRP: C-reactive protein
CT: Computed tomography
DCA: Decision curve analysis
ESR: Erythrocyte sedimentation rate
FC: Fecal calprotectin
FDA: Food and Drug Administration
IBD: Inflammatory bowel disease
ICC: Intraclass correlation coefficient
ROC: Receiver operating characteristic
ROI: Region of interest
SAI: Subcutaneous adipose index
SAT: Subcutaneous adipose tissue
SMI: Skeletal muscle index
UC: Ulcerative colitis
VAI: Visceral adipose index
VAT: Visceral adipose tissue
