# Supplementary material for: Computed tomography-based body composition parameters can predict short-term prognosis in ulcerative colitis patients
Source: Insights Imaging. 2024 Feb 27;15:60. doi: 10.1186/s13244-024-01615-w (PMC10899140; doi:10.1186/s13244-024-01615-w)

**Computed tomography-based body composition parameters can predict short-term prognosis in ulcerative colitis patients**

**ELECTRONIC SUPPLEMENTARY MATERIAL**

**Supplementary Table 1** The specific number of patients about the different levels of vertebra chosen to define the region of interest (ROI) across different disease locations.

| Level of vertebra | E1 | E2 | E3 | Total |
| --- | --- | --- | --- | --- |
| L2 | 0 | 2 | 8 | 10 |
| L3 | 13 | 36 | 63 | 102 |
| L4 | 0 | 5 | 11 | 16 |
| Total | 13 | 43 | 82 | 138 |

L2, L3 and L4: the second, third and fourth level of vertebra; E1: proctosigmoiditis; E2: left-side colitis; E3: pancolitis.

**Supplementary Table 2** The scanner parameters of the four computed tomography scanners

| CT scanners | GE  Optima CT660 | Philips Brilliance iCT 256 | GE  Revolution CT | Canon  Aquilion one |
| --- | --- | --- | --- | --- |
| Source | Center 1 | Center 1 | Center 1 | Center 2 |
| Scanner parameters |  |  |  |  |
| Tube voltage, kvp | 120 | 120 | 120 | 120 |
| Tube current, mA | 80-500 | 60-290 | 10-490 | 50-150 |
| Slice thickness, mm | 1.25 | 1.5 | 1.25 | 1.0 |
| Scan pitch ratio | 1.375:1 | 0.914:1 | 0.992:1 | 0.813:1 |
| Reconstruction diameter,mm | 376 | 406 | 350 | 404 |
| Spacing between slices, mm | 1.25 | 1.5 | 1.25 | 1.0 |

**Supplementary Table 3** The intra- and interclass correlation coefficient of computed tomography-based body composition parameters

| Imaging parameters | ICC (95%CI)  Radiologist 1 time 1 vs time 2 | ICC (95%CI)  Radiologist 1vs Radiologist 2 |
| --- | --- | --- |
| SMA surface | 0.996(0.984-0.999) | 0.986(0.960-0.991) |
| VAT surface | 0.995(0.998-0.999) | 0.965(0.912-0.989) |
| SAT surface | 0.998(0.996-0.999) | 0.996(0.988-0.999) |
| Muscle density | 0.988(0.950-0.997) | 0.953(0.901-0.976) |
| VAT density | 0.935(0.737-0.984) | 0.917(0.802-0.965) |
| SAT density | 0.981(0.922-0.995) | 0.971(0.914-0.985) |

SMA: area of skeletal muscle; VAT: visceral adipose tissue; SAT: subcutaneous adipose tissue; ICC: intra- and interclass correlation coefficient. The interval between time 1 and time 2 is one month.

**Supplementary Table 4** Diagnostic performance of the prediction model to identify patients who could not achieve rapid remission from conventional therapy in different subgroups.

| Subgroups | Acc  (%) | Sen  （%） | Spe  （%） | AUC (95%CI) | *p* value |
| --- | --- | --- | --- | --- | --- |
| E1 (n=13) | 90.30 | 87.50 | 90.00 | 0.875(0.713-0.975) | <0.001 |
| E2 (n=43) | 79.07 | 73.91 | 80.00 | 0.820(0.672-0.920) | <0.001 |
| E3 (n=82) | 78.05 | 94.29 | 65.96 | 0.855(0.760-0.923) | <0.001 |
| Center 1 (n=107) | 81.31 | 98.11 | 62.96 | 0.857(0.776-0.917) | <0.001 |
| Center 2 (n=31) | 83.87 | 84.6 | 83.30 | 0.842(0.666-0.947) | <0.001 |
| CT1 (n=12) | 90.67 | 87.5 | 90.00 | 0.869(0.687-0.969) | <0.001 |
| CT2 (n=37) | 81.08 | 94.1 | 70.00 | 0.859(0.705-0.951) | <0.001 |
| CT3 (n=58) | 81.03 | 67.9 | 93.30 | 0.871(0.757-0.945) | <0.001 |
| CT4 (n=31) | 83.87 | 84.6 | 83.30 | 0.842(0.666-0.947) | <0.001 |

E1: proctosigmoiditis; E2: left-side colitis; E3: pancolitis. Acc: accuracy; Sen: sensitivity; Spe: specificity; 95% CI: 95% confidence interval. The p value is the significance level of comparison of the AUC with that of random case (AUC=0.05). The *p* < 0.05 was identified to be of statistically significance. Numbers in the parentheses represent the number of corresponding patients.

**Supplementary Fig. 1** Flow chart of patients’ enrollment. UC: ulcerative colitis.


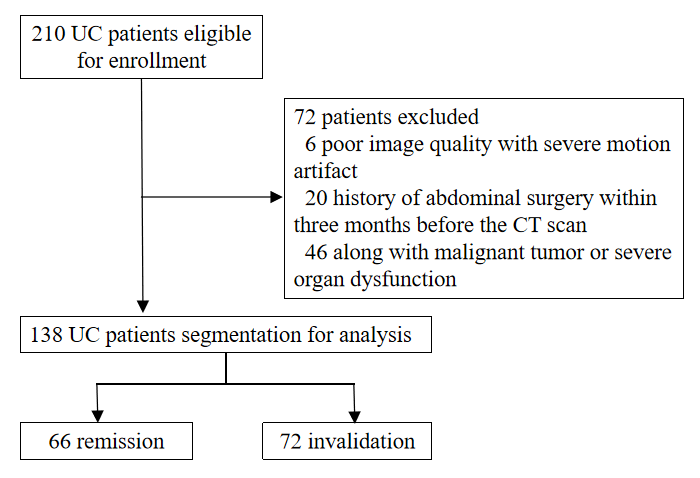

Supplement: Supplementary file 1 — Additional file 1: Table S1. The specific number of patients about the different levels of vertebra chosen to define the region of interest (ROI) across different disease locations. L2, L3 and L4: the second, third and fourth level of vertebra; E1: proctosigmoiditis; E2: left-side colitis; E3: pancolitis. Table S2. The scanner parameters of the four computed tomography scanners. Table S3. The intra- and interclass correlation coefficient of computed tomography-based body composition parameters. SMA: area of skeletal muscle; VAT: visceral adipose tissue; SAT: subcutaneous adipose tissue; ICC: intra- and interclass correlation coefficient. The interval between time 1 and time 2 is one month. Table S4. Diagnostic performance of the prediction model to identify patients who could not achieve rapid remission from conventional therapy in different subgroups. E1: proctosigmoiditis; E2: left-side colitis; E3: pancolitis. Acc: accuracy; Sen: sensitivity; Spe: specificity; 95% CI: 95% confidence interval. The p value is the significance level of comparison of the AUC with that of random case (AUC = 0.05). The p < 0.05 was identified to be of statistically significance. Numbers in the parentheses represent the number of corresponding patients. Fig. S1. Flow chart of patients’ enrollment. UC: ulcerative colitis. [file 13244_2024_1615_MOESM1_ESM.docx]
